# Sleep Disturbance Correlates with Cognitive Impairment Partly via Plasma p-Tau217: ADNI Cross-Sectional Evidence and P301L Mouse Mechanistic Observations

**DOI:** 10.3390/ijms27188271

**Published:** 2026-09-17

**Authors:** Qiong Zheng, Run-Shuang Liu, Bei-Rui Chen, Ai-Ling Chen, Qiu-Cheng Zhu, Hao Zhang, Zhuo-Ying Chen, Xiang-Jie Liu

**Affiliations:** 1 Li-Yuan Hospital, Tongji Medical College, Huazhong University of Science and Technology, Wuhan 430077, China; zq13026382296@163.com (Q.Z.); 17724512016@163.com (R.-S.L.); chenbeirui2026@163.com (B.-R.C.); m202476909@hust.edu.cn (A.-L.C.); 18337328327@163.com (Q.-C.Z.); 13971129375@163.com (H.Z.); 2 Laboratory of Metabolic Abnormalities and Vascular Aging, Huazhong University of Science and Technology, Wuhan 430077, China

**Keywords:** Alzheimer’s disease, sleep disturbance, tau hyperphosphorylation, cognitive impairment

## Abstract

How sleep disturbances link to cognitive decline in Alzheimer’s disease (AD) via tau pathology is unclear. We explored whether sleep dysfunction worsens cognitive deficits by elevating plasma phosphorylated tau217 (p-Tau217). We combined Alzheimer’s Disease Neuroimaging Initiative (ADNI) human cohort mediation analysis with 6-week chronic sleep deprivation (SD) assays in P301L tau transgenic mice. Out of 5423 screened subjects, we included 230 participants with complete data. Clinical analyses revealed sleep disturbance independently linked to higher plasma p-Tau217 and worse cognition. Bootstrap mediation confirmed p-Tau217 partially mediated the sleep–cognition link, accounting for a 44.8% mediating effect. This pathway only existed in mild cognitive impairment (MCI) and AD patients. We also ruled out reverse causality. In mice, chronic SD triggered broad cognitive deficits without obvious stress elevation. Behavioral dysfunction was accompanied by hippocampal neuronal loss, glial overactivation, and widespread p-Tau217 hyperphosphorylation, whereas total tau showed only mild, sex-restricted upregulation. These animal observations corroborated our clinical results. In conclusion, we characterize a stage-specific correlational sleep-p-Tau217-cognition axis associated with early AD pathological changes. These findings provide preliminary translational clues linking sleep disruption to p-Tau217 dysregulation and subsequent cognitive impairment, laying a foundation for future exploration of sleep-targeted strategies for early AD intervention.

## 1. Introduction

Sleep disturbances have become a global public health issue, with their prevalence steadily increasing, particularly in aging societies. Epidemiological evidence indicates that sleep disturbances are not only risk factors for cardiovascular disease and psychiatric disorders but are also closely associated with cognitive decline [1,2,3]. As the most common neurodegenerative disease of the central nervous system, AD is pathologically characterized by extracellular β-amyloid (Aβ) deposition forming senile plaques and intraneuronal aggregation of hyperphosphorylated tau protein forming neurofibrillary tangles [4]. In recent years, plasma phosphorylated tau at threonine 217 (p-Tau217) has been recognized as a promising blood biomarker for AD due to its excellent sensitivity and specificity [5], accurately reflecting changes in brain Aβ and tau pathology [6]. Therefore, identifying and elucidating how modifiable risk factors (e.g., sleep disorders) accelerate cognitive decline by affecting key pathological pathways is crucial for early prevention and intervention of AD.

Currently, numerous studies have revealed a complex association between sleep disorders and the risk of dementia [7], yet the underlying biological mechanisms remain to be elucidated. Although sleep deprivation has been confirmed to increase brain Aβ production and deposition, its impact on tau pathology, particularly phosphorylated tau, still lacks sufficient clinical evidence and causal inference. Preclinical studies suggest that sleep disruption may upregulate tau phosphorylation levels through activation of stress pathways [8]. In AD patients, plasma p-Tau217 levels are not only positively correlated with the degree of cognitive impairment but also closely associated with heterogeneity in disease progression [6]. Nevertheless, large clinical studies rarely ask whether sleep disorders directly elevate p-Tau217 and thereby harm cognition. No previous work has measured the exact pathway connecting sleep issues to cognitive decline through p-Tau217. The absence of such mechanistic data restricts efforts to build lifestyle-targeted intervention plans for the early stages of AD pathology.

Therefore, this study aims to investigate whether sleep disturbances exert an indirect effect on cognitive function by elevating plasma p-Tau217. We used clinical data from the ADNI database. First, we analyzed the baseline characteristics across different diagnostic groups (cognitively normal [CN], MCI, and AD) and sleep status groups. Subsequently, we used multiple linear regression models to check the independent effects of sleep disturbances and p-Tau217 on cognition. Next, we ran a bootstrap-based mediation analysis. This tested if plasma p-Tau217 bridges the gap between sleep disturbances and cognitive function. Finally, we grouped the data by disease stage to see if this mechanism changes over time.

We also ran animal experiments to test the biology behind this clinical finding. We selected the P301L tau transgenic mouse model, which spontaneously develops tau hyperphosphorylation and aggregation, serving as a classic tool for studying tau pathology [9,10,11]. We subjected them to a 6-week sleep intervention using an automated sleep deprivation device. This setup mimicked human chronic sleep disturbance [12]. Subsequently, we systematically assessed hippocampal-dependent cognitive behavior, neuroinflammatory responses, and tau hyperphosphorylation levels.

This study adopts a translational medicine strategy combining “clinical data analysis and animal experimental validation,” aiming to provide a complete mechanistic evidence chain for the phenomenon of “sleep disturbance exacerbating cognitive decline in AD.” In the first part, we elaborate on the clinical statistical analysis from the ADNI database. In the second part, we explain the animal experimental methods, covering the construction of a chronic sleep deprivation model, behavioral tests, histopathological and molecular biological assays. Ultimately, we aim to confirm the plasma p-Tau217 is the key molecular bridge linking sleep disturbances to cognitive impairment, which provides a scientific basis for treating early AD by improving sleep.

## 2. Results

### 2.1. ADNI Database Analysis

#### 2.1.1. Participant Screening and Analysis Workflow

**Figure 1:**
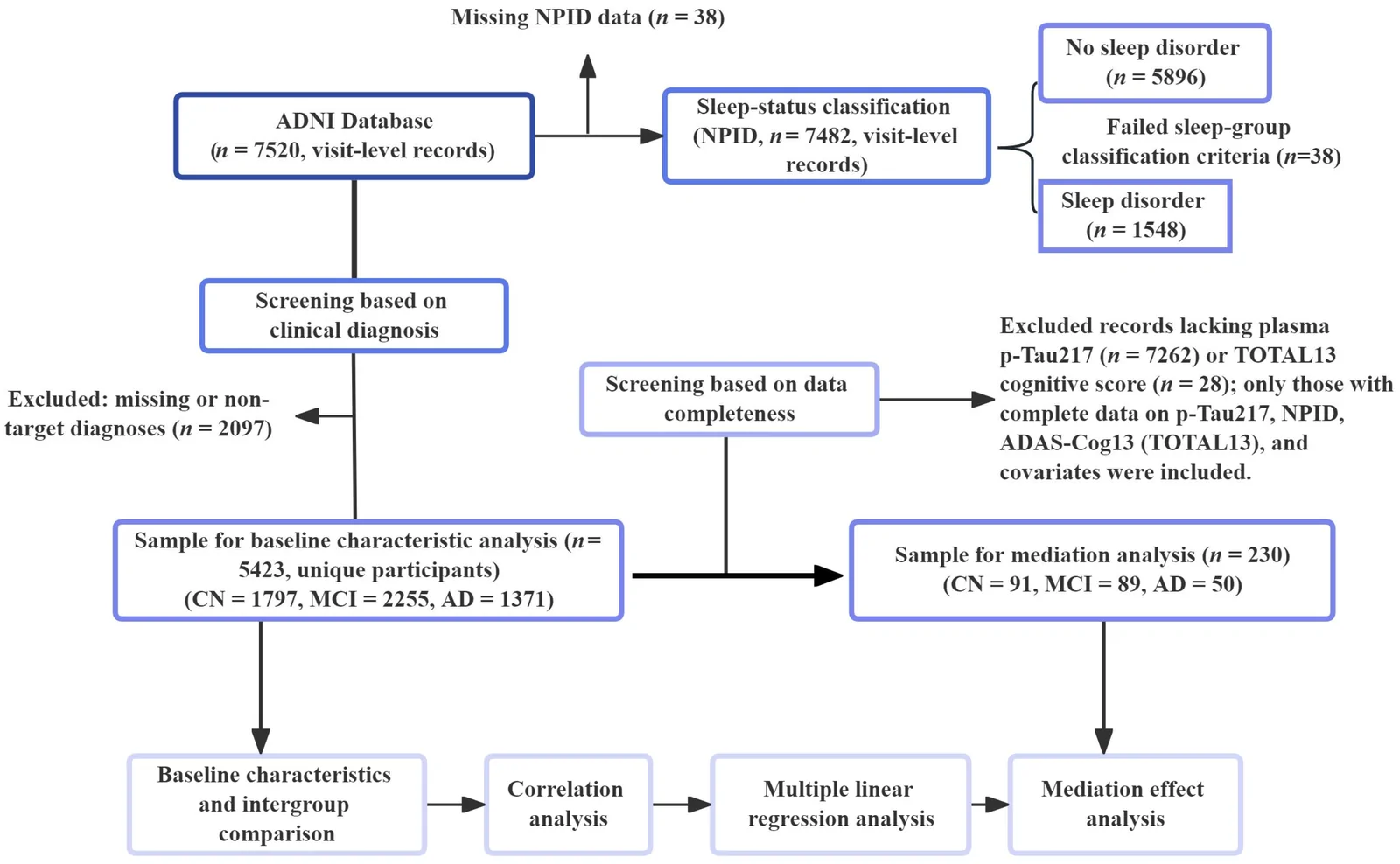
ADNI database analysis workflow.

This study was based on the ADNI database, initially including a total of 7520 visit-level observation records (rather than independent unique participants). According to the different research objectives, sample screening followed two independent paths, and stratified analyses were performed (Figure 1).

First, sleep-status classification was performed at the visit-record level: out of the initial 7520 visit-level records, 7482 records contained valid and complete NPID sleep-disturbance data. Among these 7482 records, a further 38 records could not be assigned to either sleep-related group, because their NPID scores did not meet our predefined binary classification criteria (0 = no sleep disturbance, 1 = presence of sleep disturbance). After removing these unclassifiable records, the final sample for sleep-stratified comparison included 7444 records: 5896 in the no-sleep-disturbance group and 1548 in the sleep-disturbance group. Records were then screened by clinical diagnosis, and those with missing or non-target diagnoses (*n* = 2097) were excluded. This ultimately yielded 5423 eligible unique participants: the CN group (*n* = 1797), the MCI group (*n* = 2255), and the AD group (*n* = 1371). This sample was used for baseline characterization, between-group comparisons across diagnostic groups, correlation analysis and multiple-linear-regression analyses.

Second, for the mediation analysis, screening was performed based on the completeness of core-variable data across the initial 7520 visit-level records. Visit-level records lacking plasma p-Tau217 data (*n* = 7262) and those with missing TOTAL13 cognitive scores (*n* = 28) were excluded. The remaining records with complete data were further restricted to participants belonging to the 5423 clinically qualified baseline cohort. Finally, a total of 230 visit-level records with complete data on p-Tau217, NPID, cognitive scores, and covariates were included in the final mediation analysis. This analysis sample comprised 91 participants in the CN group, 89 in the MCI group, and 50 in the AD group.

Based on these two samples, the study sequentially performed baseline characterization and inter-group comparisons, correlation analysis, and multiple linear regression analysis, and ultimately applied a mediation model to explore the mechanism by which sleep disturbance affects cognitive function through plasma p-Tau217. Notably, some of the 7262 visit-level records excluded for missing plasma p-Tau217 data had already been removed during the clinical diagnosis-screening phase of the baseline-characterization path. The final mediation-analysis sample (*n* = 230) came from participants who met all inclusion criteria.

#### 2.1.2. Baseline Characteristics Analysis Results

Table 1 presents the baseline characteristics stratified by diagnostic group. A total of 7520 initial visit-level records were extracted from the ADNI database. Among these records, 5423 unique participants had confirmed diagnostic labels, comprising CN participants (*n* = 1797), individuals with MCI (*n* = 2255), and patients with AD (*n* = 1371). Of these 5423 unique participants, 230 visit-level records contained complete data for all core variables and were used for subsequent mediation-effect analyses.

Compared with the CN and MCI groups, participants in the AD group were older and had fewer years of education (all *p* < 0.05). The AD group showed the highest *APOE* ε4 carrier frequency (67.3%), plasma p-Tau217 levels, NPID, and the highest ADAS-Cog13 cognitive score, indicating the most severe cognitive impairment (all *p* < 0.001). All corresponding indicators in the MCI group were intermediate between those of the CN and AD groups. The Aβ_42_/Aβ_40_ ratio also differed significantly among the three diagnostic groups (*p* < 0.001); post hoc testing showed that AD participants exhibited a significantly lower Aβ_42_/Aβ_40_ ratio than CN participants (adjusted *p* < 0.001), whereas no significant differences were found for the CN-MCI (adjusted- *p* = 0.094) and MCI-AD (adjusted *p* = 0.567) comparisons. The total sample was further stratified by NPID into a non-sleep disturbance group (*n* = 5896) and a sleep disturbance group (*n* = 1548). This comparison is presented in Table 2. The *APOE* ε4 carrier rate was significantly higher in the sleep disturbance group than in the non-sleep disturbance group (54.7% vs. 41.8%, *p* < 0.001), and the ADAS-Cog13 cognitive score was higher (*p* < 0.001), suggesting more severe cognitive impairment. A small but statistically significant difference in age was observed between the two groups (*p* = 0.002), and the Aβ_42_/Aβ_40_ ratio also differed significantly by sleep disturbance status *(p =* 0.031). In summary, baseline analysis revealed differential patterns of demographic, genetic, and pathological characteristics across disease progression and sleep disturbance status. Notably, sleep disturbance was associated with a higher *APOE* ε4 risk, higher p-Tau217 levels, and worse cognitive function, whereas its association with Aβ pathology (Aβ_42_/Aβ_40_) was weaker. These findings provide a basis for subsequent exploration of the mediating role of p-Tau217 in the relationship between sleep disturbance and cognitive function.

#### 2.1.3. Correlation Analysis

**Figure 2:**
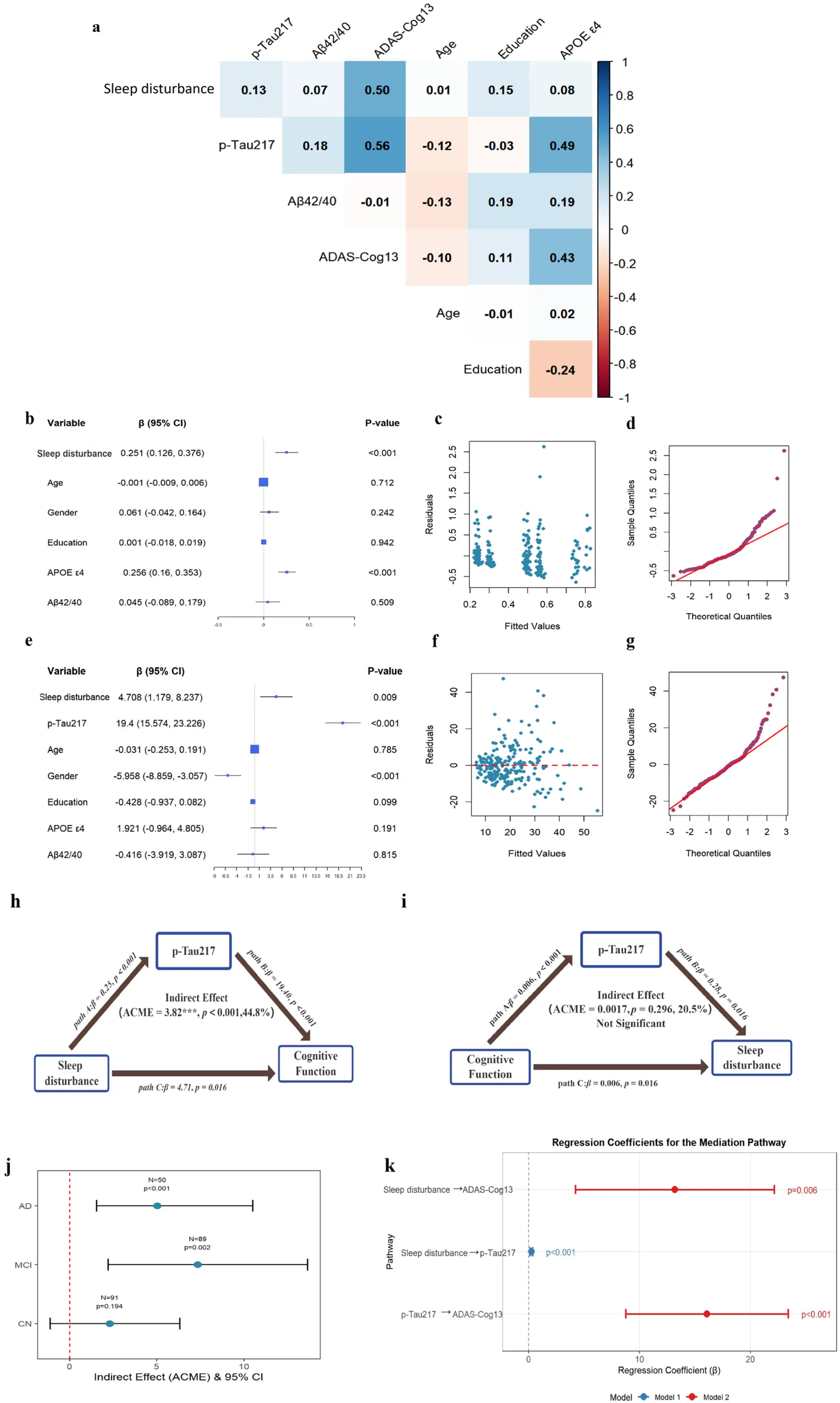
Correlation, Multiple Regression, and Mediation Effect Analysis in the ADNI Cohort (*n* = 230): (**a**) Heatmap of bivariate correlations among core study variables. (**b**) Forest plot of regression coefficients for Model 1 (sleep disturbance predicting plasma p-Tau217). (**c**) Residual scatter diagnostic plot for Model 1. (**d**) Residual Q-Q normal distribution plot for Model 1. (**e**) Forest plot of regression coefficients for Model 2 (sleep disturbance and p-Tau217 jointly predicting ADAS-Cog13 cognitive scores). (**f**) Residual scatter diagnostic plot for Model 2. (**g**) Residual Q-Q normal distribution plot for Model 2. (**h**) Schematic diagram of the forward mediation pathway (sleep disturbance → p-Tau217 → cognitive impairment). (**i**) Schematic diagram of the reverse mediation pathway (cognitive impairment → p-Tau217 → sleep disturbance). (**j**) Comparison of subgroup mediation effects across CN/MCI/AD strata. (**k**) Forest plot of path coefficients summarizing the mediation analysis across the full sample. Red dashed lines in panels (**f**,**j**) represent the reference line at zero. *** *p* < 0.001.

To preliminarily characterize the associations among sleep disturbance, AD-related biomarkers, genetic risk factors, and cognitive function, bivariate correlation analyses were performed on *n* = 230 visit-level records after completeness screening of core study variables. Pearson correlation was used for pairs of normally distributed continuous variables; Spearman rank-order correlation was applied for non-normally distributed continuous variables, and point-biserial correlation was adopted to test associations between binary variables (NPID, *APOE* ε4 carrier status) and continuous variables. The results were visualized in a correlation heatmap (Figure 2a), where blue indicates positive correlations and red indicates negative correlations.

The correlation results showed that the severity of sleep disturbance (NPID, the sleep-disturbance sub-score of the Neuropsychiatric Inventory) was moderately and significantly positively correlated with the ADAS-Cog13 cognitive impairment score (*r* = 0.50, *p* < 0.001), suggesting that greater sleep disturbance is associated with more severe cognitive deficits. Sleep disturbance severity showed only a weak, non-significant positive correlation with plasma p-Tau217 (*r* = 0.13, *p* = 0.392), and a very weak, non-significant correlation with the plasma Aβ_42_/Aβ_40_ ratio (*r* = 0.07, *p* = 0.671).

Plasma p-Tau217 levels exhibited a weak positive, non-significant correlation with the Aβ_42_/Aβ_40_ ratio (*r* = 0.18, *p* = 0.250). This within-sample bivariate association does not contradict the well-described negative pathological trend observed across CN/MCI/AD diagnostic strata; however, as this correlation did not reach statistical significance, we avoid further biological over-interpretation. Plasma p-Tau217 also showed a significant positive correlation with APOE ε4 carrier status (*r* = 0.49, *p* < 0.001). The ADAS-Cog13 cognitive score was significantly positively correlated with plasma p-Tau217 levels (*r* = 0.56, *p* < 0.001) and *APOE* ε4 carrier status (*r* = 0.43, *p* = 0.004). Years of education was weakly and non-significantly negatively correlated with *APOE* ε4 carrier status (*r* = −0.24, *p* = 0.114). Age showed very low correlation coefficients with all other variables, with no significant statistical associations, suggesting minimal confounding interference from age on the core-variable associations in this study.

In summary, the correlation analysis directly revealed a stable positive-association gradient among sleep disturbance, plasma p-Tau217, *APOE* ε4 genotype, and cognitive impairment, preliminarily identifying plasma p-Tau217 as a potential mediating target. These findings provide a statistical basis for subsequent multiple regression analyses adjusting for confounders and for constructing mediation models.

#### 2.1.4. Multiple Linear Regression Analysis

To adjust for the confounding effects of age, sex, education, *APOE* genotype, and Aβ levels, and to determine the independent contributions of sleep disturbance and plasma p-Tau217 to cognitive function, two sets of hierarchical multiple linear regression models were constructed. Model diagnostics were performed using residual scatter plots and Q-Q normal probability plots.

Model 1 treated plasma p-Tau217 level as the dependent variable, with the sleep disturbance index as the primary independent variable, adjusting for age, sex, years of education, *APOE* ε4 carrier status, and the plasma Aβ_42_/Aβ_40_ ratio (Figure 2b). The regression results showed that sleep disturbance independently and positively predicted plasma p-Tau217 levels (*β* = 0.251, 95% CI: 0.126–0.376, *p* < 0.001). *APOE* ε4 carrier status was also an independent risk factor for elevated plasma p-Tau217 (*β* = 0.256, 95% CI: 0.160–0.353, *p* < 0.001). Age, sex, years of education, and the Aβ_42_/Aβ_40_ ratio showed no independent predictive effect on p-Tau217 (all *p* > 0.05). Residual diagnostics for Model 1 (Figure 2d) revealed a random and uniform distribution of residuals, and the Q-Q plot aligned closely with the theoretical normal distribution, satisfying the assumptions of normality and homoscedasticity for linear regression, confirming the reliability of the model fit.

Model 2 took the ADAS-Cog13 cognitive score as the outcome variable, simultaneously including the sleep disturbance index and plasma p-Tau217 as independent variables, while adjusting for the same set of covariates as in Model 1 (Figure 2e). The results indicated that both sleep disturbance (*β* = 4.708, 95% CI: 1.179–8.237, *p* = 0.009) and plasma p-Tau217 level (*β* = 19.400, 95% CI: 15.574–23.226, *p* < 0.001) were independent risk factors for cognitive decline. Sex exhibited an independent protective effect (*β* = −5.958, *p* < 0.001), with female participants showing relatively less cognitive impairment. The plasma Aβ_42_/Aβ_40_ ratio had no independent influence on the cognitive score (*p* = 0.815), suggesting that cognitive impairment in this population was primarily driven by tau pathology. Corresponding residual diagnostics for Model 2 (Figure 2f,g) displayed no obvious clustering or trend in the residuals, and the Q-Q plot points closely followed the diagonal reference line, confirming that Model 2 also met the assumptions of linear regression and that the regression results were robust and reliable.

In summary, the multiple linear regression analyses demonstrated that sleep disturbance could independently upregulate peripheral plasma p-Tau217 levels and, in concert with p-Tau217, independently and synergistically exacerbate cognitive decline.

#### 2.1.5. Mediation Analysis

To clarify the role of plasma p-Tau217 in the association between sleep disturbance and cognitive function, A bootstrap-based mediation analysis was performed. The ACME was used to quantify the magnitude of the mediating effect. The results of the positive mediation model are shown in Figure 2h: sleep disturbance exhibited a significant direct effect on cognitive impairment (Path C: *β* = 4.71, *p* = 0.016), along with a significant positive indirect mediation effect (ACME = 3.82, *p* < 0.001), with the indirect effect accounting for 44.8% of the total effect. These findings indicate that plasma p-Tau217 partially mediates the statistical association between sleep disturbance and cognitive decline, with nearly half of the negative effect of sleep on cognition being exerted through promoting tau phosphorylation.

To examine reverse causality, a reverse mediation model was constructed (with cognitive function as the independent variable, p-Tau217 as the mediator, and sleep disturbance as the outcome; Figure 2i). The indirect effect in the reverse model was not statistically significant (ACME = 0.0017, *p* = 0.296, proportion of indirect effect = 20.5%), indicating that the reverse directional pathway “cognitive decline → p-Tau217 → sleep disturbance” was not statistically supported; given the cross-sectional design, this does not definitively establish sleep disturbance as an upstream causal factor.

Subgroup mediation analyses stratified by disease diagnosis are shown in Figure 2j. This mediation pathway exhibited clear disease-stage specificity: the mediation effect was significant in the AD group (*n* = 50, *p* < 0.001) and in the MCI group (*n* = 89, *p* = 0.002), but not in the CN group (*n* = 91, *p* = 0.194). This suggests that this statistical association is detectable only in participants with established neurodegeneration (MCI and AD stages) and is not observed in cognitively normal older adults. A forest plot of path coefficients (Figure 2k) intuitively displays the regression coefficients and significance levels for the three core pathways, fully consistent with the decomposition of the mediation effects.

In summary, the mediation analysis supports our preset hypothesis of this study: sleep disturbance may mediate cognitive decline by elevating plasma p-Tau217 levels, with a mediating contribution of 44.8% of the total effect, and this statistical association is observed only in the MCI and AD stages. These findings provide population-level evidence supporting sleep intervention as a strategy to delay cognitive decline in AD.

The above cross-sectional ADNI cohort analysis indicates a statistical associative pattern between sleep disturbance, plasma p-Tau217 and cognitive impairment, which is only evident in MCI and AD participants. Subsequently, in vivo intervention experiments were conducted using 3-month-old P301L tau transgenic mice to further validate, at the hippocampal histological level, the intrinsic pathological mechanisms by which chronic sleep deprivation regulates tau phosphorylation, induces sex-specific neuroinflammation, and causes granule neuron injury. This achieves a bidirectional corroboration between clinical macro-level associations and animal micro-level histopathological mechanisms.

### 2.2. Chronic Sleep Deprivation Induces Widespread Cognitive Deficits in P301L Mice

**Figure 3:**
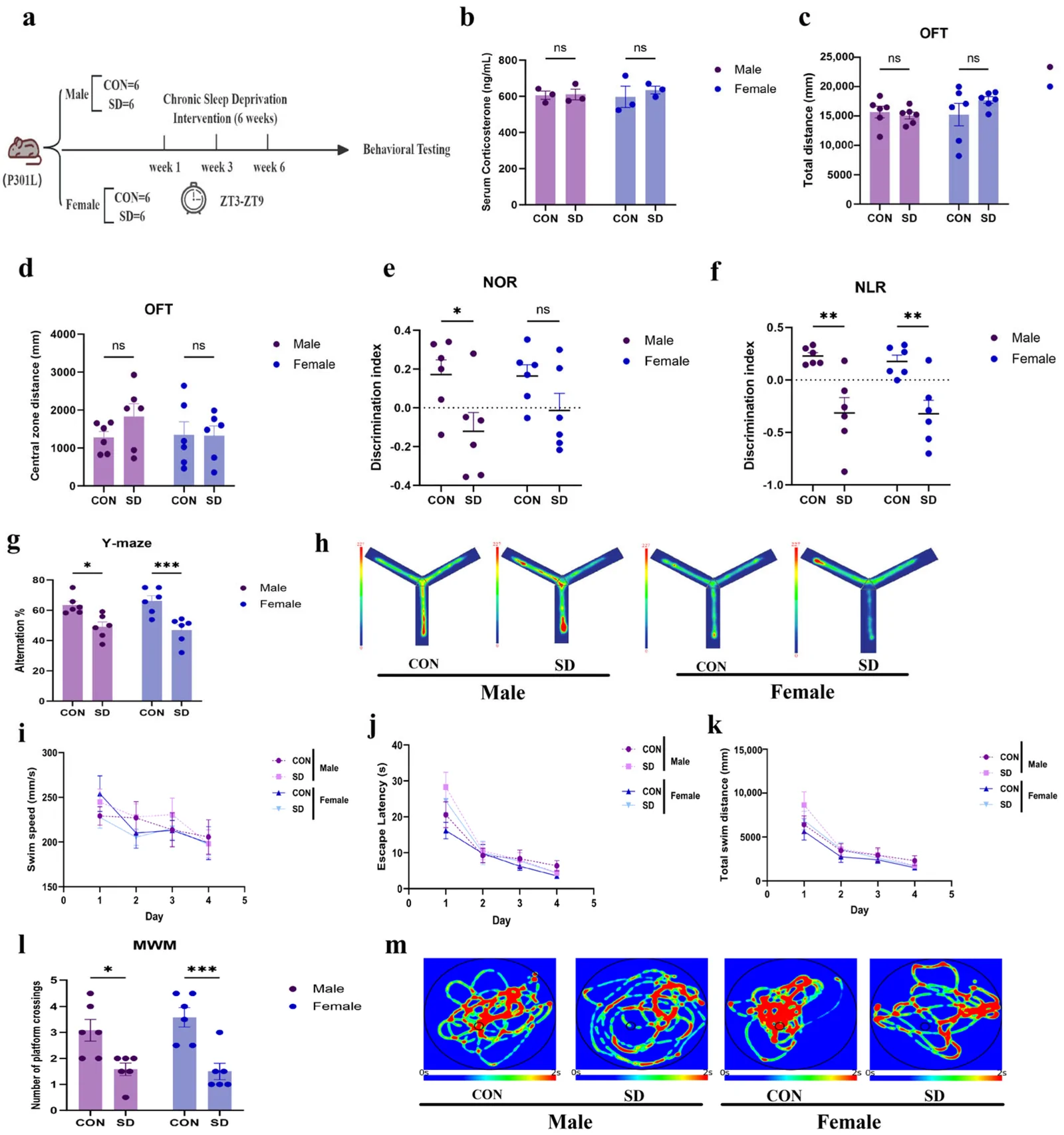
Chronic sleep deprivation induces widespread cognitive deficits in P301L mice. (**a**) Schematic diagram of the 6-week chronic sleep-deprivation intervention (ZT3-ZT9) and subsequent behavioral test schedule. For male and female P301L mice, *n* = 6 per group for all behavioral assays, except for serum corticosterone measurement in panel. (**b**) Quantification of serum corticosterone levels (*n* = 3). (**c**) Total travel distance measured in the OFT. (**d**) Central-zone travel distance in the OFT, representing anxiety-like behavior. (**e**) Discrimination index of the NOR test. (**f**) Discrimination index of the NLR test. (**g**,**h**) Y-maze working-memory assessment. (**g**) Spontaneous alternation percentage; (**h**) Representative residence heatmaps of Y-maze trajectories for male and female mice. (**i**–**k**) Swimming speed, escape latency and total swim distance recorded during 4 consecutive days of MWM acquisition training. (**l**) Number of crossings over the removed target platform. Two separate probe trials were performed on day 5 (24 h) and day 6 (48 h) after the final acquisition-training session, and the mean value from the two trials was used for statistical analysis. (**m**) Representative swimming trajectory heatmaps of male and female mice in the MWM probe trial. Data from panels (**b**–**h**,**l**,**m**) were analysed using two-way ANOVA followed by pairwise comparison. Repeated-measures two-way ANOVA was used for longitudinal training-curve data in panels (**i**–**k**). ns, not significant; * *p* < 0.05, ** *p* < 0.01, *** *p* < 0.001.

To exclude the confounding effects of general locomotor dysfunction and elevated stress as potential drivers of cognitive impairment, we first performed OFT and detected serum corticosterone levels in male and female P301L mice subjected to 6 weeks of chronic sleep deprivation (SD) and corresponding control (CON) littermates (Figure 3a).

In OFT, two-way ANOVA revealed no significant main effects of sleep intervention (Row factor) or sex (Column factor), and no interaction between the two factors for total travel distance (Interaction *p* = 0.1961, sleep *p* = 0.4230, sex *p* = 0.3568; Figure 3c) and central zone exploration distance (Interaction *p* = 0.3349, sleep *p* = 0.3706, sex *p* = 0.4553; Figure 3d). Consistently, serum corticosterone concentration showed no statistical differences across all four experimental groups (Interaction *p* = 0.6657, sleep *p* = 0.5784, sex *p* = 0.8293; Figure 3b). These data indicated that chronic SD did not alter spontaneous locomotor ability, anxiety-like behavior, or systemic glucocorticoid stress response in P301L mice, eliminating these confounding variables for subsequent cognitive behavioral assessments.

We next evaluated short-term recognition memory via NOR and NLR paradigms. For NOR discrimination index (Figure 3e), two-way ANOVA identified a significant main effect of sleep deprivation (*F* = 8.442, DF = 1,20, *p* = 0.0087), with no significant sex effect or interaction between sex and sleep treatment. Post hoc pairwise comparison demonstrated a markedly reduced discrimination index in SD mice relative to CON counterparts within both male and female subgroups, indicating disrupted novel object recognition memory following sleep loss. Similarly, the NLR yielded a robust main effect of chronic SD on spatial location discrimination index (*F* = 25.78, DF = 1,20, *p* < 0.0001; Figure 3f), while sex and interaction terms remained non-significant. SD mice exhibited substantially impaired preference for relocated objects, revealing compromised short-term spatial recognition memory independent of biological sex.

Spatial working memory was assessed using the Y-maze spontaneous alternation assay (Figure 3g). Two-way ANOVA confirmed a prominent main effect of sleep intervention (*F* = 27.24, DF = 1,20, *p* < 0.0001), with no significant sex difference or interaction effect. Alternation percentage was significantly lower in SD mice compared to CON mice in both sexes, demonstrating that chronic sleep deprivation impairs intact working memory function in P301L transgenic mice. Representative residence heatmaps of Y-maze trajectories further illustrated exploration patterns (Figure 3h). CON mice distributed activity evenly across all three arms, whereas SD mice showed biased residence concentrated within a single arm, consistent with reduced alternation rates in quantitative analysis.

The MWM was applied to probe long-term spatial reference memory (Figure 3i–l). During the 4-day acquisition phase, two-way repeated-measures ANOVA was performed for escape latency, total swim distance and swimming speed. No significant interaction between sleep intervention and sex was observed for escape latency (*F* (9, 60) = 1.224, *p* = 0.2980), total swim distance (*F* (9, 60) = 0.6199, *p* = 0.7754) and swimming speed (*F* (9, 60) = 0.3251, *p* = 0.9635). A significant main effect of sleep deprivation was detected for all three training indices (escape latency: *F* (3, 20) = 35.89, *p* < 0.0001; total swim distance: *F* (3, 20) = 35.74, *p* < 0.0001; swim speed: *F* (3, 20) = 5.171, *p* = 0.0083), whereas the main effect of sex remained non-significant. Visual inspection of learning curves revealed comparable declining trends across all experimental groups during training (Figure 3i–k). On day 5 (24 h) and day 6 (48 h) after the last acquisition-training session, two independent MWM probe trials (without the hidden platform) were conducted. The average number of crossings over the former target platform across the two probe tests was calculated and analysed. Probe-trial quantification (Figure 3l) demonstrated a significant main effect of chronic SD (*F* = 27.27, DF = 1,20, *p* < 0.0001), without significant sex or interaction effects. SD mice crossed the original target platform region far fewer times than sex-matched CON animals, as visualized by representative swimming trajectory heatmaps (Figure 3m). Collectively, these results demonstrated that 6-week chronic sleep deprivation induced measurable long-term spatial memory changes in P301L mice, independent of sex differences and general swimming ability.

Across NOR, NLR, Y-maze, and MWM assays, chronic sleep deprivation consistently impaired short-term recognition memory, working memory, and long-term spatial reference memory in both male and female P301L mice. Given the absence of changes in basal locomotion, anxiety-like behavior, and circulating corticosterone levels, these widespread cognitive deficits are attributed to sleep loss itself rather than secondary alterations in general activity or stress signaling. No significant interaction between sleep intervention and sex was detected for any cognitive endpoint measured, suggesting comparable susceptibility to SD-induced memory impairment between male and female P301L mice.

### 2.3. Chronic Sleep Deprivation Triggers Hippocampal Neuronal Loss and Reactive Gliosis in P301L Mice

Given the cognitive impairments detected in our behavioral tests, we next performed histological and immunofluorescence staining on mouse hippocampal tissue, to explore potential structural and glial pathological alterations induced by chronic sleep deprivation [13].

**Figure 4:**
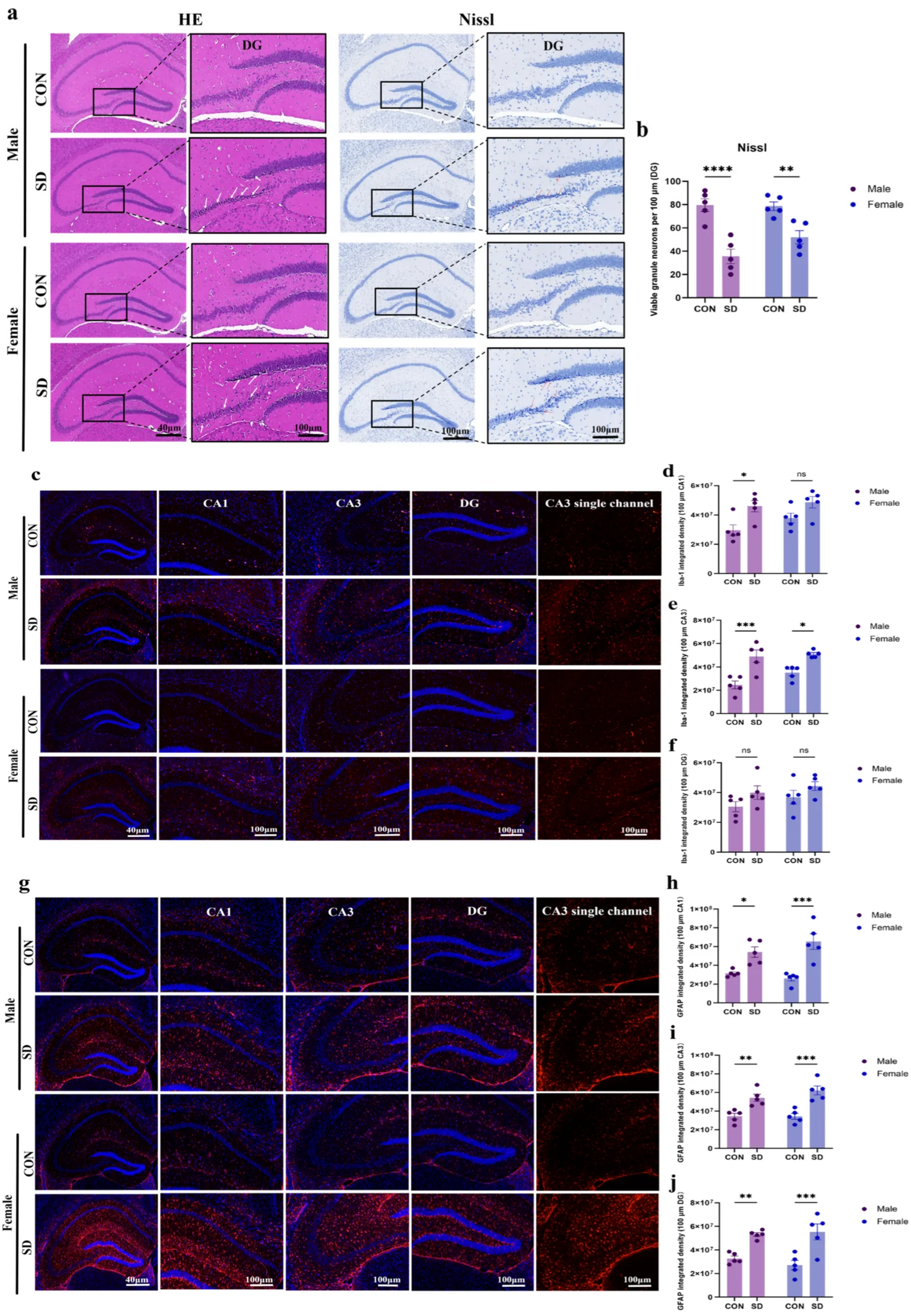
Chronic Sleep Deprivation Triggers Hippocampal Neuronal Loss and Reactive Gliosis in P301L Mice. (**a**) Representative hippocampal H&E and Nissl staining images of male and female CON and SD mice, focusing on the DG region (*n* = 5). (**b**) Quantification of viable Nissl-positive granule neurons per 100 μm DG granule cell layer. (**c**–**f**) Iba-1 immunofluorescence for microglia (*n* = 5); red indicates Iba-1, blue indicates DAPI nuclear counterstain. (**c**) Representative multichannel and CA3 single-channel images of CA1, CA3 and DG subregions; (**d**–**f**) Quantification of Iba-1 integrated fluorescence density in CA1, CA3 and DG, respectively. (**g**–**j**) GFAP immunofluorescence for astrocytes (*n* = 5); red indicates GFAP, blue indicates DAPI nuclear counterstain. (**g**) Representative multichannel and CA3 single-channel images of CA1, CA3 and DG subregions; (**h**–**j**) Quantification of GFAP integrated fluorescence density in CA1, CA3 and DG, respectively. All quantitative data were analyzed using two-way ANOVA followed by pairwise comparisons; ns, not significant; * *p* < 0.05, ** *p* < 0.01, *** *p* < 0.001, **** *p* < 0.0001.

We conducted H&E and Nissl staining to evaluate neuronal morphological features and the number of intact neurons within the dentate gyrus (DG) (Figure 4a). Under HE staining, we noticed irregular neuronal nuclear morphology in the DG granule cell layer of SD mice, compared with neatly arranged intact neurons in control animals. Nissl staining further allowed us to quantify Nissl-positive viable granule neurons (Figure 4b). We analyzed the neuronal count data using two-way ANOVA, and we identified a significant main effect of sleep intervention (*F* = 43.73, DF = 1,16, *p* < 0.0001). We detected no significant main effect of sex, nor a significant interaction between sex and sleep treatment. Post hoc pairwise comparisons showed lower counts of viable DG granule neurons in SD mice relative to sex-matched CON littermates, in both male (**** *p* < 0.0001) and female subgroups (** *p* < 0.01). These observations indicate chronic sleep deprivation correlates with fewer intact DG granule neurons in P301L mice, regardless of biological sex.

We carried out Iba-1 immunofluorescence staining to label microglia across the CA1, CA3 and DG hippocampal subfields (Figure 4c), and we calculated integrated fluorescence density to reflect microglial status in each region. In the CA1 subfield (Figure 4d), two-way ANOVA revealed a significant main effect of sleep deprivation (*F* = 13.51, DF = 1,16, *p* = 0.0020), while sex and interaction terms did not reach statistical significance. We observed higher Iba-1 integrated density in male SD mice (*p* < 0.05), whereas we found no meaningful difference between female CON and SD groups. In the CA3 subfield (Figure 4e), we detected a significant main effect of sleep intervention (*F* = 34.10, DF = 1,16, *p* < 0.0001). Post hoc tests showed increased Iba-1 signal intensity in both male (*** *p* < 0.001) and female (* *p* < 0.05) SD mice. For the DG region (Figure 4f), we found no statistically significant differences in Iba-1 density between CON and SD groups for either sex. Together, these results suggest chronic sleep deprivation is associated with elevated microglial signal specifically within CA1 and CA3, with limited microglial response detected in the DG granule layer.

We performed GFAP immunofluorescence staining to examine astrocytic profiles in CA1, CA3 and DG subfields (Figure 4g), and we quantified GFAP integrated density for each region: For CA1 (Figure 4h): Two-way ANOVA showed a significant main effect of sleep deprivation (*F* = 34.20, DF = 1,16, *p* < 0.0001). We recorded higher GFAP fluorescence density in male (*p* < 0.05) and female (*** *p* < 0.001) SD mice relative to matched controls. For CA3 (Figure 4i): A significant main effect of sleep intervention was identified (*F* = 39.10, DF = 1,16, *p* < 0.0001). GFAP signal was elevated in male (** *p* < 0.01) and female (*** *p* < 0.001) SD mice. For DG (Figure 4j): Two-way ANOVA also yielded a significant main effect of chronic sleep deprivation (*F* = 34.38, DF = 1,16, *p* < 0.0001). We observed higher GFAP density in male (** *p* < 0.01) and female (*** *p* < 0.001) SD groups. We did not observe significant main effects of sex or interaction between sex and sleep treatment for GFAP measurements in any hippocampal subregion. These data demonstrate chronic sleep deprivation correlates with increased astrocytic GFAP signal throughout CA1, CA3 and DG in both male and female P301L mice.

Collectively, our histological and immunofluorescence assays identified three measurable changes linked to 6-week chronic sleep deprivation in P301L mice: reduced numbers of viable DG granule neurons, region-specific elevation of microglial signal within CA1 and CA3, and increased astrocytic GFAP signal across all tested hippocampal subfields. All measured pathological readouts were primarily driven by sleep intervention rather than biological sex, consistent with the comparable cognitive changes we observed in male and female SD mice in our preceding behavioral tests.

### 2.4. Sleep Deprivation Increases Hippocampal p-Tau217 (Thr217) Expression

**Figure 5:**
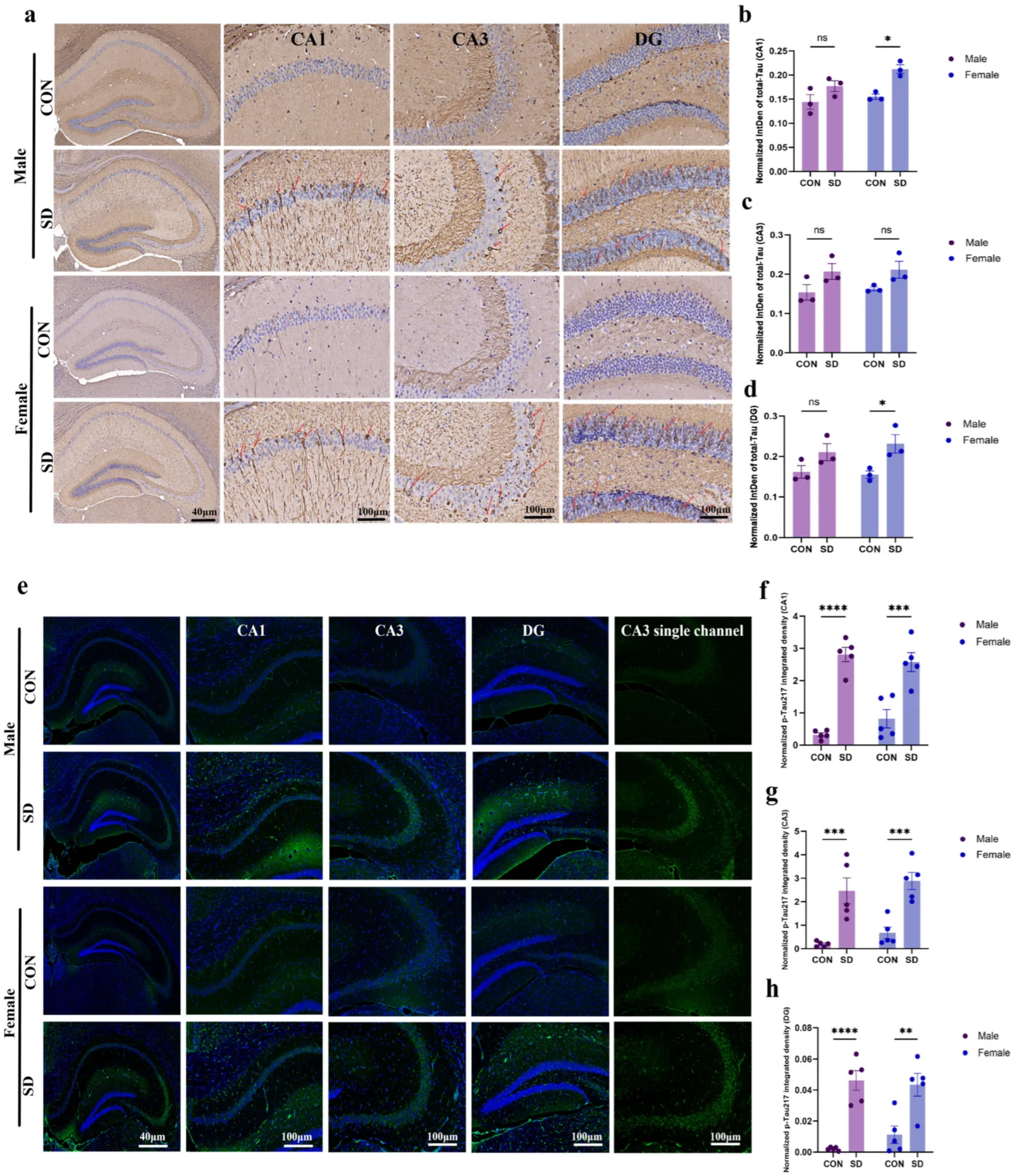
Sleep deprivation increases hippocampal p-Tau217 (Thr217) expression. (**a**) Representative immunohistochemical staining of total Tau within hippocampal CA1, CA3 and DG subregions in control (CON) and sleep deprivation (SD) mice (*n* = 3). Red arrows indicate total-Tau-positive cells. (**b**–**d**) Quantitative analysis of normalized integrated density of total Tau immunohistochemical signals in CA1, CA3 and DG. (**e**) Representative confocal immunofluorescence micrographs for p-Tau217 (green), with DAPI nuclear counterstain (blue). Low-magnification hippocampal overview (scale bar = 40 μm) and magnified single subregion views (CA1, CA3, DG, CA3 single-channel; scale bar = 100 μm) are presented (*n* = 5). (**f**–**h**) Quantification of normalized integrated fluorescence density of p-Tau217 in CA1, CA3 and DG. All quantitative data were analyzed using two-way ANOVA followed by pairwise comparisons; ns, not significant; * *p* < 0.05, ** *p* < 0.01, *** *p* < 0.001, **** *p* < 0.0001.

Consistent with the neuronal atrophy and glial activation detected by Nissl, H&E and GFAP staining, we next quantified total Tau and p-Tau217, a pathological Tau epitope linked to neurofibrillary tangles, across hippocampal CA1, CA3 and DG after chronic sleep deprivation (SD). We carried out total Tau immunohistochemistry (Figure 5a) and calculated normalized integrated density to assess Tau deposition. In CA1 (Figure 5b), two-way ANOVA identified a significant main effect of SD (*F* = 17.28, DF = 1,8, *p* = 0.0032); sex and interaction effects were non-significant. Bonferroni post hoc tests showed elevated total Tau only in female SD mice (* *p* < 0.05), with no change in males. In CA3 (Figure 5c), SD exerted a significant main effect (*F* = 8.017, DF = 1,8, *p* = 0.0221), yet no sex- or treatment-dependent pairwise differences were found between CON and SD groups of either sex. In DG (Figure 5d), SD yielded a significant main effect (*F* = 12.14, DF = 1,8, *p* = 0.0083); post hoc comparisons revealed increased total Tau solely in female SD mice (* *p* < 0.05). Collectively, total Tau deposition exhibited mild, female-restricted upregulation without pan-hippocampal accumulation across all animals.

We further performed p-Tau217 immunofluorescence (Figure 5e) and quantified its normalized integrated density to evaluate pathological Tau hyperphosphorylation. In CA1 (Figure 5f), two-way ANOVA confirmed a robust main effect of SD (*F* = 82.49, DF = 1,16, *p* < 0.0001), while sex and interaction terms were insignificant. Post hoc tests showed markedly higher p-Tau217 in both male (**** *p* < 0.0001) and female (*** *p* < 0.001) SD mice. In CA3 (Figure 5g), only SD exerted a significant main effect (*p* < 0.0001); both male and female SD mice displayed elevated p-Tau217 signals. In DG (Figure 5h), SD produced a significant main effect (*F* = 46.13, DF = 1,16, *p* < 0.0001), with no sex or interaction effect. p-Tau217 fluorescence was significantly increased in SD mice of both sexes. These data demonstrate chronic SD induces widespread Tau hyperphosphorylation at Thr217 across all three hippocampal subregions, independent of sex baseline differences.

In summary, total Tau and p-Tau217 exhibited divergent responses to SD in P301L mouse hippocampus. While total Tau only rose moderately in female mice within partial subregions, p-Tau217 was universally upregulated in all SD animals regardless of sex. The lack of significant sex-by-treatment interaction for p-Tau217 indicates sleep loss acts as an independent upstream trigger of disease-relevant Tau hyperphosphorylation. These results provide quantitative evidence linking disrupted sleep homeostasis to the early pathological accumulation of phosphorylated Tau.

## 3. Discussion

AD is the most common type of dementia, characterized pathologically by extracellular deposition of β-amyloid (Aβ) plaques and intracellular neurofibrillary tangles formed by hyperphosphorylated tau protein [4,14,15]. Although the core pathological mechanisms of AD have been extensively studied, the modulatory factors driving its progression remain incompletely understood. Sleep disturbance, a highly prevalent comorbid condition, is increasingly recognized as a potential risk factor and early prodromal marker for neurodegenerative diseases [16,17,18,19,20]. Clinical evidence indicates that disruption of the sleep–wake cycle not only affects cognitive function [21,22] but is also closely associated with the development of various neurological disorders, including AD.

Martin et al. (2024) have already reported male-specific cognitive vulnerability and accelerated tau pathology induced by chronic sleep deprivation in PS19 mice, findings that are highly consistent with the sex-specific effects we observed [13]. Additionally, Holth et al. established progressive tau-driven EEG/sleep disruption in this family of models [23], while the work of Vetter et al. and Irmen et al. further explored the direct link between sleep disruption and tau burden [24,25]. Together, these findings constitute an important background for our study. However, our work extends prior preclinical findings by providing cross-sectional human-cohort evidence for the statistical associative pattern of the “sleep-p-Tau217-cognition” pathway using minimally invasive plasma biomarkers. This provides a translational bridge from basic mechanistic observations to clinical correlative evidence. Therefore, we propose a potential “sleep-p-Tau217-cognition axis”. Although inspired by prior pre-clinical work, this hypothesis generates novel insights based on correlational evidence from our human cohort. Further interventional studies are required to establish whether p-Tau217 acts as a mediator linking sleep disturbance to cognitive decline. In future work, we will more thoroughly explore the connections and differences between these foundational studies and our human-cohort findings.

Accumulating studies have validated a bidirectional regulatory loop linking sleep homeostasis to Aβ clearance: sleep loss impairs glymphatic drainage of Aβ and triggers cerebral amyloid accumulation; reciprocally, Aβ plaque deposition disrupts sleep regulatory nuclei and exacerbates sleep fragmentation, forming an amplifying vicious cycle [26,27]. However, Aβ primarily drives pathological alterations in preclinical AD and mainly mediates early synaptic toxicity and neural network hyperexcitability, whereas tau hyperphosphorylation directly correlates with cognitive decline in MCI and advanced AD [28,29,30]. Consistent with this stage-specific pathological pattern, our regression analyses revealed that after adjusting for confounders, the plasma Aβ_42_/Aβ_40_ ratio barely correlated with p-Tau217 and failed to independently predict cognitive impairment. These results suggest that tau pathology, rather than Aβ, may be more strongly associated with sleep-related cognitive differences across AD-spectrum patients with measurable cognitive deficits.

Multiple lines of evidence support plasma p-Tau217 as the core mediating biomarker in this work. First, tau hyperphosphorylation emerges earlier in memory circuits including the entorhinal cortex and hippocampus than cortical Aβ plaque formation, which simultaneously explains prodromal sleep disturbances and incipient cognitive deterioration [31]. Second, phosphorylated tau directly disrupts microtubule stability and impairs synaptic plasticity, acting as a direct effector molecule for neuronal dysfunction [32]. Third, plasma p-Tau217 is highly specific, minimally invasive, and partially reflective of cerebral tau burden; compared with cerebrospinal fluid (CSF) testing and PET imaging, it is more suitable for mechanistic validation in large population cohorts [33,34]. Notably, all regression and mediation models in this study were adjusted for plasma Aβ_42_/Aβ_40_ ratio and *APOE* ε4 genotype. Meanwhile, our animal experiments adopted P301L mice that only express mutant human tau without spontaneous Aβ plaque formation. Together, cross-sectional clinical and interventional animal data are consistent with an Aβ-independent statistical “sleep-p-Tau217-cognition” associative pattern.

Combining the ADNI clinical cohort and chronic sleep-deprived P301L mouse model, this study systematically explored how sleep disturbance regulates cognitive function via p-Tau217. Cohort analyses demonstrated that plasma p-Tau217 exerted a significant mediating effect on the association between sleep disturbance and cognitive impairment, accounting for 44.8% of the total effect. This pathological pathway was only evident in MCI and AD participants, but absent in CN individuals, implying stage-specific pathogenicity. Animal histological and behavioral results further demonstrated that chronic sleep deprivation in P301L mice triggered hippocampal tau hyperphosphorylation, neuronal injury and cognitive deficits, providing mechanistic corroboration of the direction suggested by the human cross-sectional data.

Notably, the raw Pearson correlation coefficient between sleep disturbance severity and plasma p-Tau217 only reached *r* = 0.13, representing a trivial linear bivariate association that could hardly independently support the direct linear “sleep-p-Tau217-cognition” cascade observed in simple correlation analysis. Despite this weak zero-order correlation, the mediation model still identified a significant indirect mediating effect accounting for 44.8% of the total effect of sleep disturbance on cognitive impairment. This apparent discrepancy between weak bivariate correlation and robust mediation effect can be explained by the essential difference between simple pairwise correlation and pathway-based mediation analysis: Pearson correlation merely reflects crude linear association between two single variables without adjusting for co-existing AD pathological confounders (e.g., Aβ_42_/Aβ_40_ ratio, *APOE* ε4 status), while mediation modeling quantifies the indirect transmitted effect of sleep disturbance on cognitive decline specifically through p-Tau217 after partitioning total effects into direct and indirect pathways.

Mechanistically, sleep disturbance activates tau kinases such as glycogen synthase kinase-3β (GSK-3β) and cyclin-dependent kinase 5 (CDK5) to boost tau hyperphosphorylation, destabilize neuronal microtubules and accelerate neurofibrillary tangle formation [35,36,37]. Our animal assays confirmed elevated hippocampal tau phosphorylation after sleep deprivation, matching the upregulated plasma p-Tau217 observed in human subjects. Furthermore, sleep deprivation induced robust hippocampal neuroinflammation characterized by massive activation of reactive microglia and astrocytes. Excessive glial activation releases abundant pro-inflammatory cytokines to construct a chronic neuroinflammatory microenvironment, which synergistically aggravates tau pathology and neuronal degeneration [38,39]. Cell-level experimental results showed that sleep deprivation impaired object recognition and spatial memory, accompanied by reduced survival of hippocampal granule cells and chromatolysis of Nissl bodies. Intriguingly, female mice exhibited more severe spatial memory deficits following sleep loss, suggesting sex hormones may modulate tau pathogenicity. After adjusting for Aβ and genetic confounders, plasma p-Tau217 independently predicted cognitive decline in clinical participants. This suggests that sleep disturbance may represent an active risk factor associated with accelerated tau pathological signaling and cognitive decline in AD-spectrum populations.

This study has several limitations. First, incomplete plasma p-Tau217 data limited the cohort sample size (*n* = 230), which may reduce statistical power and restrict the generalizability of our findings to broader populations. Second, reverse causality tests supported our hypothesized directional pathway, yet the cross-sectional design of ADNI data cannot fully establish strict temporal causal relationships between variables. Third, sleep disturbance was solely assessed via caregiver-rated NPI sleep subscores, without objective tools including polysomnography and actigraphy, nor standardized sleep questionnaires such as the Pittsburgh Sleep Quality Index (PSQI) and Epworth Sleepiness Scale; this may introduce reporting bias. Fourth, our clinical mediation analyses were based on circulating plasma p-Tau217, whereas animal validation only examined hippocampal tissue p-Tau217 expression. In addition, we did not perform genetic or pharmacological inhibition of Thr217 tau phosphorylation to verify the causal role of p-Tau217 in sleep disturbance-induced cognitive impairment. Accordingly, current preclinical results can only support correlational associations rather than confirm causal mediation effects. Fifth, all animal experiments were conducted in P301L tau-transgenic mice without age-matched wild-type controls, which cannot clarify whether increased p-Tau217 is a specific pathological response to AD-related tau pathology or a nonspecific general molecular alteration induced by sleep deprivation. Sixth, P301L mice predominantly recapitulate tau pathology without robust Aβ deposition, and thus cannot fully recapitulate the complex multifactorial pathological landscape of human AD. Finally, sex-stratified mediation analyses were not performed on clinical data; future large-sample research is required to further dissect sex-specific pathogenic mechanisms.

In conclusion, this study integrates human-cohort-based mediation analysis and P301L transgenic animal observation to identify a correlational “sleep-p-Tau217-cognition” pathological pathway associated with early AD progression. Mediation models revealed a statistical correlational pattern, suggesting that plasma p-Tau217 may be involved in the cross-sectional association between sleep disturbance and cognitive impairment among patients across the AD cognitive spectrum. Our in vivo mouse data further verified that chronic sleep deprivation triggers hippocampal microglial activation, selective Tau hyperphosphorylation at Thr217, and neuronal atrophy, which correlate with disrupted hippocampus-dependent cognitive function. Collectively, our results identify plasma p-Tau217 as a minimally invasive translational biomarker correlated with sleep loss and pre-clinical cognitive decline. Notably, given the cross-sectional clinical design and lack of targeted p-Tau217-intervention and wild-type animal validation in the present study, causal inference and therapeutic implications should be interpreted cautiously. Future interventional and mechanistic studies are required to further confirm the causal pathogenic cascade and translational value of the sleep-p-Tau217 axis.

## 4. Material and Methods

### 4.1. ADNI Database

#### 4.1.1. Ethics Statement

This study conducted a secondary retrospective analysis of the public ADNI database, strictly adhering to the data use guidelines. Original participants gave written informed consent. The overall research protocol was approved by the ethics committees of each collaborating center. This study only used de-identified, anonymized public data for statistical analysis, involving no identifiable personal privacy information and posing no risk of privacy breach. Therefore, no additional ethical approval or secondary informed consent was required.

#### 4.1.2. Study Population and Baseline Characteristics

The data of this study were obtained from the ADNI database (https://adni.loni.usc.edu, accessed on 1 May 2026) [40,41]. The initial dataset contained 7520 visit-level observation records, which represent repeated follow-up assessments and do not correspond one-to-one to unique individual participants. Records with missing or non-target clinical diagnoses (*n* = 2097) were excluded from the 7520 visit-level records, yielding 5423 unique participants with confirmed diagnostic labels, including CN (*n* = 1797), MCI (*n* = 2255), and AD (*n* = 1371) individuals. Demographic variables such as sex were summarized based on the full set of 7520 visit-level records.

Sleep-stratified analyses were conducted at the visit-record level using the Neuropsychiatric Inventory sleep-disturbance sub-score (NPID). Among all visit-level records, 7482 had complete NPID data. Of these, 38 records could not be assigned to binary sleep groups because their NPID scores did not meet our predefined classification criteria (0 = no sleep disturbance; 1 = presence of sleep disturbance). After excluding these unclassifiable entries, the sleep-stratified analytical dataset comprised 7444 visit-level records: 5896 records in the no-sleep-disturbance group and 1548 records in the sleep-disturbance group.

For mediation analysis, complete data for plasma p-Tau217, ADAS-Cog13 (TOTAL13) cognitive scores, and all pre-specified covariates were required. We first screened across the full initial 7520 visit-level records and excluded 7262 records lacking plasma p-Tau217 measurements and 28 records with missing TOTAL13 cognitive scores. Remaining records with complete variables were further restricted to those belonging to the diagnostically qualified pool of 5423 unique participants. This procedure produced the final mediation-analysis dataset of 230 visit-level records, consisting of 91 CN, 89 MCI, and 50 AD records.

#### 4.1.3. Study Variable Selection

We summarized the baseline demographic, genetic, clinical, cognitive, and sleep-related variables in Table S1. Demographic characteristics included age, sex, and years of education. The genetic risk factor was defined by *APOE* ε4 genotype, classified as *APOE* ε4 carrier or non-carrier. Core AD-associated biomarkers comprised plasma phosphorylated tau217 (p-tau217) levels and the plasma amyloid-β 42/40 (Aβ_42_/Aβ_40_) ratio. Global cognitive function was evaluated using the TOTAL13 score, with the ADAS-Cog13 score additionally applied for correlation analyses. Sleep disturbance severity was assessed based on the NPID scale. All variables were rigorously screened for subsequent statistical modeling and correlation analysis.

#### 4.1.4. Statistical Analysis of the ADNI Cohort

All statistical analyses were performed using R (version 4.5.2) with two-sided tests, and a *p* value < 0.05 was considered statistically significant.

##### Descriptive Statistics and Group Comparisons

Continuous variables were first tested for normality using the Shapiro–Wilk test. Variables following a normal distribution were presented as mean ± standard deviation (Mean ± SD), with group comparisons performed using one-way analysis of variance (ANOVA) and post hoc pairwise comparisons using Tukey’s HSD test. Variables not following a normal distribution were presented as median (interquartile range) [M (IQR)], with group comparisons performed using the Kruskal–Wallis H test and post hoc pairwise comparisons using Dunn’s test with Bonferroni correction. Categorical variables were presented as frequency (percentage) [n (%)], with group comparisons performed using the chi-square test. Measurements of the plasma Aβ_42_/Aβ_40_ ratio in the ADNI dataset included a small number of extreme outlying values. Primary analyses were based on rank-based nonparametric tests, which are robust to outliers.

##### Correlation Analysis

To explore the associations among sleep disturbance, plasma biomarkers, and cognitive function, bivariate correlation analyses were performed on *n =* 230 visit-level records after completeness screening of core study variables. The sleep-disturbance variable (NPID) was dichotomized as follows: 0 = no sleep disturbance, 1 = sleep disturbance. APOE ε4 carrier status was also coded as a binary variable: 1 = ε4 carrier (including the ε3/ε4 and ε4/ε4 genotypes), 0 = non-carrier (including ε2/ε2, ε2/ε3, ε3/ε3). Participants with missing or invalid *APOE* genotype data were excluded from correlation analysis. Pearson correlation was used for pairs of normally distributed continuous variables. Spearman rank-order correlation was used for continuous variables that deviated from normality. Point-biserial correlation (statistically equivalent to Pearson correlation) was applied to associations between binary variables (NPID, APOE ε4 carrier status) and continuous outcome variables. Full variable coding definitions are provided in Table S2 for reproducibility.

##### Multiple Linear Regression Analysis

To investigate the independent and combined effects of sleep disturbance and plasma p-Tau217 on cognitive function, two multiple linear regression models were constructed on *n* = 230 visit-level records after completeness screening of core study variables. Both models were adjusted for age, sex, years of education, *APOE* ε4 genotype, and the Aβ_42_/Aβ_40_ ratio. Model 1: plasma p-Tau217 level was entered as the dependent variable and the sleep disturbance index as the independent variable. Model 2: ADAS-Cog13 (the corresponding raw variable name within the ADNI database: TOTAL13) cognitive score was entered as the dependent variable, with both the sleep disturbance index and plasma p-Tau217 level included as independent variables. Multicollinearity was assessed using the variance inflation factor (VIF < 10 considered acceptable). The normality and homoscedasticity of model residuals were examined using quantile–quantile (Q-Q) plots and scatterplots of residuals versus fitted values.

##### Mediation Analysis

A bootstrap-based mediation analysis was conducted on *n* = 230 visit-level records after completeness screening of core study variables to test the potential mediating role of plasma p-Tau217 in the association between sleep disturbance and cognitive function. The sleep disturbance index was used as the independent variable (X), plasma p-Tau217 was the mediator (M), and the TOTAL13 score was the dependent variable (Y), adjusting for the aforementioned covariates. Using the R package *mediation*, 1000 Bootstrap resamples were drawn to estimate the average causal mediation effect (ACME), average direct effect (ADE), and total effect. An indirect effect was considered statistically significant if its 95% confidence interval (CI) did not include zero. Reverse-causality testing and sensitivity analyses were additionally performed—(i) increasing the number of bootstrap resamples to 2000, (ii) excluding the Aβ_42_/Aβ_40_ covariate, and (iii) removing outliers beyond ±3 standard deviations—to verify the robustness of the results and the direction of causality.

##### Stratified Mediation Analysis

The same causal mediation model as in the primary analysis was applied within each diagnostic subgroup: the independent variable was NPID, the mediator was plasma p-Tau217 level, the dependent variable was the TOTAL13 cognitive score, and covariates included age, sex, years of education, *APOE* ε4 genotype, and the Aβ_42_/Aβ_40_ ratio. The ACME for each subgroup was estimated using 1000 nonparametric bootstrap resamples to compute bias-corrected 95% CIs. An indirect effect was considered statistically significant if its 95% CI did not include zero.

### 4.2. Animal Experiment

#### 4.2.1. Experimental Animals and Ethical Approval

In this study, P301L Tau mutant transgenic mice were used to establish an AD pathological model. This mutation drives abnormal Tau phosphorylation and aggregation. At 3 months of age, the mice slowly begin to develop early p-Tau deposition, gradually forming neurofibrillary tangles, closely mimicking the characteristic Tau pathology of AD. At this age, only mild baseline pathology is present, representing an early intervention window. The 6-week sleep deprivation intervention was designed to reveal Tau accumulation, glial activation, and neuronal injury induced solely by sleep loss, without interference from severe spontaneous pathology associated with aging. Tau pathology in this model preferentially affects the entorhinal cortex–hippocampus circuitry. These mice also exhibit intrinsic sex differences in Tau pathology and glial activation, making them suitable for sex-stratified grouping in this study. Moreover, the model primarily exhibits Tau pathology without substantial spontaneous Aβ plaques, thereby eliminating Aβ interference and allowing clear delineation of the causal pathway through which sleep deprivation drives Tau hyperphosphorylation, neuroinflammation, and neuronal injury.

The mice were divided into four groups: male control (Male CON), male sleep deprivation (Male SD), female control (Female CON), and female sleep deprivation (Female SD), with *n* = 6 mice per group. The housing environment was maintained at 22 ± 2 °C, humidity 50% ± 10%, with a 12 h light/dark cycle and ad libitum access to food and water. The animal experiment protocol was approved by the Laboratory Animal Welfare and Ethics Committee of Huazhong University of Science and Technology (Ethics Approval No.: SY20260349). All animal interventions, tissue collection, and euthanasia procedures strictly complied with national and institutional regulations on laboratory animal welfare.

#### 4.2.2. Chronic Sleep Deprivation Intervention

An automated chronic sleep deprivation apparatus (Model RS-SL-02, Guangzhou Hongshi Scientific Instrument Co., Ltd, Guangzhou, China) equipped with a sweeping bar was used. The device was set to rotate forward for 3 s, pause for 5 s, and rotate in reverse for 3 s. The apparatus gently touched mice about to enter sleep via a slowly rotating platform or soft sweeping bar, preventing them from achieving sustained sleep while avoiding stress or physical injury. The intervention lasted for 6 weeks, conducted daily during the photoperiod from ZT3 to ZT9 [12]. Control group mice were placed in the same environment without activating the deprivation procedure.

#### 4.2.3. Behavioral Testing Methods

Following the 6-week chronic sleep deprivation intervention, all mice underwent a series of behavioral tests in a fixed sequence. All tests were performed in a blinded manner (the experimenter was unaware of group allocation) to minimize observer bias. The testing environment, lighting, and noise levels were kept consistent. Mice were acclimated to the behavioral testing room for 24 h before testing began. All behavioral video data were automatically collected and analyzed using an animal behavior tracking system [42].

##### Open Field Test (OFT)

Testing was conducted using a closed open-field apparatus measuring 40 cm × 40 cm × 30 cm. Each mouse was placed in the central zone and allowed to freely explore for 5 min. The system automatically recorded the total distance traveled, the time spent in the central zone, and the number of entries into the central zone to evaluate spontaneous locomotor activity and anxiety-like behavior.

##### Novel Object Recognition Test (NOR)

The novel object recognition test was used to assess non-spatial learning and memory ability in mice. The experiment consisted of three phases: a familiarization phase, an inter-trial interval, and a test phase. During the familiarization phase, two identical objects were placed symmetrically in the apparatus, and the mouse was allowed to freely explore for 5 min. This was followed by a 24-h inter-trial interval, during which the mouse was returned to its home cage for normal rest. During the test phase, one of the objects was replaced with a novel object differing in shape and color, and the mouse was allowed to freely explore for 5 min. The exploration time for the novel and familiar objects was recorded, and the discrimination index (DI) was calculated as: DI = (time exploring the novel object − time exploring the familiar object)/(time exploring the novel object + time exploring the familiar object).

##### Novel Location Recognition Test (NLR)

This test was used to assess spatial location memory in mice. The experiment consisted of three phases: a familiarization phase, an inter-trial interval, and a test phase. During the familiarization phase, two identical objects were placed symmetrically in the apparatus, and the mouse was allowed to freely explore for 5 min. This was followed by a 24-h inter-trial interval, during which the mouse was returned to its home cage for normal rest. During the test phase, the objects remained unchanged in appearance, but one object was moved to a novel location. The mouse was allowed to freely explore for 5 min. The exploration time for objects in the novel and familiar locations was recorded, and the discrimination index (DI) was calculated as: DI = (time exploring the object in the novel location − time exploring the object in the familiar location)/(time exploring the object in the novel location + time exploring the object in the familiar location). This index was used to evaluate spatial recognition memory in the mice.

##### Y-Maze Test

The mouse was placed at the starting point in the central arm of the Y-maze and allowed to freely explore for 5 min. The sequence of arm entries and the total number of entries were recorded throughout the session. Spontaneous alternation rate was calculated using the formula: Spontaneous alternation rate (%) = [Number of alternations/(Total arm entries − 2)] × 100%. This measure reflects short-term spatial working memory ability in mice.

##### Morris Water Maze Test

The Morris water maze test was used to assess long-term spatial learning and memory consolidation in mice. The experiment consisted of a place navigation phase and a spatial probe phase. The place navigation phase lasted for 4 days, during which a hidden platform was fixed in the target quadrant. Mice were placed into the water from different starting points each day, and the escape latency, swimming speed, and total swimming distance were recorded to evaluate spatial learning ability. On days 5 and 6, the platform was removed, and two spatial probe trials were conducted. The number of times the mouse crossed the original platform location was recorded to evaluate long-term spatial memory retrieval and retention.

#### 4.2.4. Serum Corticosterone ELISA

Serum corticosterone levels were quantified using a competitive corticosterone-specific ELISA kit (Cat. No. MK0525OA, Meike Biotechnology, Nanjing, China). All experimental procedures were strictly performed in accordance with the manufacturer’s instructions.

#### 4.2.5. Brain Tissue Histopathological and Molecular Biological Detection

Mice were anesthetized with isoflurane inhalation and perfused via the left ventricle with pre-cooled PBS followed by 4% paraformaldehyde (Cat. No. G1101, Servicebio, Wuhan, China) for fixation. Whole brains were collected, post-fixed in 4% paraformaldehyde for 24 h, and then subjected to gradient dehydration, clearing, and paraffin embedding. Serial coronal sections were prepared [43,44].

##### Hematoxylin and Eosin (H&E) Staining

Paraffin sections were routinely deparaffinized and rehydrated. Sections were stained with hematoxylin solution for 5 min, followed by differentiation in acid alcohol and bluing. Then, eosin solution was applied for 2 min. After gradient alcohol dehydration, clearing with xylene, and mounting with neutral resin, the overall brain morphology and neuronal structural changes were observed under a light microscope.

##### Nissl Staining

Paraffin sections were deparaffinized and rehydrated, then stained with 0.5% toluidine-blue solution (Cat. No. 71041284, Sinopharm Chemical Reagent Co., Ltd., Shanghai, China) at 60 °C for 30 min. After rinsing with distilled water, the sections were rapidly dehydrated through graded ethanol (Cat. No. 10009218, Sinopharm Chemical Reagent Co., Ltd., Shanghai, China), cleared in xylene (Cat. No. 10023418, Sinopharm Chemical Reagent Co., Ltd., Shanghai, China), and mounted with neutral balsam (Cat. No. 20200237, Nanchang Yulu Experimental Equipment Co., Ltd., Nanchang, China). The distribution and morphology of Nissl bodies in neurons were observed under a high-resolution digital pathological slide-scanning system (Pannoramic SCAN, 3DHISTECH, Budapest, Hungary) to evaluate neuronal injury.

##### Immunohistochemical Staining (Tau)

Immunohistochemistry on paraffin sections was performed to detect the expression of total-Tau protein in mouse hippocampal tissues. Sections were deparaffinized and rehydrated, followed by heat-induced antigen retrieval in citrate buffer (pH 6.0) under high temperature and pressure. Endogenous peroxidase activity was blocked with 3% H_2_O_2_, and nonspecific binding was blocked with 5% normal goat serum at room temperature for 30 min. Sections were incubated with rabbit anti-total-Tau primary antibody (Proteintech, Wuhan, China: 10274-1-AP, 1:200) overnight at 4 °C. After incubation with HRP-conjugated secondary antibody at 37 °C for 30 min, sections were visualized using DAB chromogen, counterstained with hematoxylin, dehydrated, cleared, and mounted with neutral resin. Images were captured and quantitative analysis was conducted under a light microscope.

##### Immunofluorescence Staining (Iba-1/GFAP/p-Tau217)

Paraffin sections were used for immunofluorescence detection of glial cell activation status and p-tau217 expression. Sections were deparaffinized and rehydrated, followed by antigen retrieval as described above. After permeabilization with 0.3% Triton X-100 (Immunostaining Permeabilization Buffer, Cat. No. P0096, Beyotime Biotechnology, Shanghai, China) and blocking with 5% bovine serum albumin (BSA) at room temperature, sections were incubated with primary antibodies overnight at 4 °C, including Iba-1 (Servicebio: GB15105-50, 1:400), GFAP (Proteintech, Wuhan, China: 60190-1-Ig, 1:500), and p-tau217 (Bioss, Beijing, China: BS-2843R, 1:200). Sections were then incubated with fluorescent secondary antibodies at 37 °C for 1 h in the dark. Nuclei were counterstained with DAPI (Cat. No. C1002, Beyotime Biotechnology, Shanghai, China), and sections were mounted with an anti-fade mounting medium. Images were captured and analyzed under a confocal laser scanning microscope to evaluate microglial activation, astrocyte activation, and p-tau217 expression levels.

#### 4.2.6. Statistical Analysis of Animal Experiments

Animal experimental data were analyzed with GraphPad Prism 10.1.2. Shapiro–Wilk test was used for normality verification, and data were presented as mean ± SD. Two-way analysis of variance (ANOVA) with Bonferroni’s post hoc multiple comparison test was used for behavioral metrics and histological cell count quantification. Post hoc comparisons were only performed upon significant omnibus ANOVA outcomes. Significance thresholds: ns, not significant; * *p* < 0.05, ** *p* < 0.01, *** *p* < 0.001.

## Figures and Tables

**Table 1:** Baseline characteristics of the study population stratified by diagnostic group.

Variable	CN (*n* = 1797)	MCI (*n* = 2255)	AD (*n* = 1371)	*p*-Value
Age, years	74.1 ± 6.7	74.1 ± 7.1	75.0 ± 7.3	0.001
Education, years	16.3 ± 2.6	15.8 ± 2.9	15.2 ± 3.0	<0.001
Sex, *n* (%)				<0.001
Male	881 (49.0)	1415 (62.7)	780 (56.9)	
Female	916 (51.0)	840 (37.3)	591 (43.1)	
pTau217	0.22 ± 0.20	0.43 ± 0.33	0.81 ± 0.49	<0.001
ADAS-Cog13 cognitive score	9.0 ± 4.3	17.4 ± 7.4	31.6 ± 8.1	<0.001
*APOE* ε4 carrier status, *n* (%)				<0.001
Non-carrier	1289 (71.7)	1149 (51.0)	449 (32.8)	
Carrier	508 (28.3)	1106 (49.0)	922 (67.3)	
Aβ_42_/Aβ_40_	0.0866 (0.0774, 0.0982)	0.0824 (0.0758, 0.0884)	0.0806 (0.0751, 0.0833)	<0.001
NPID sleep-disturbance index	0.09 ± 0.28	0.26 ± 0.44	0.34 ± 0.47	<0.001

Abbreviations: CN, cognitively normal; MCI, mild cognitive impairment; AD, Alzheimer’s disease. Continuous variables with a normal distribution are presented as mean ± standard deviation; non-normally distributed plasma Aβ_42_/Aβ_40_ ratio is reported as median (interquartile range). Between-group differences were tested using one-way ANOVA for normally distributed continuous variables, the Kruskal–Wallis H-test followed by Dunn’s post hoc test with Bonferroni correction for Aβ_42_/Aβ_40_ratio, and the χ^2^ test for categorical variables.

**Table 2:** Baseline characteristics of participants stratified by sleep disturbance status.

Variable	No Sleep Disturbance (*n* = 5896)	Sleep Disturbance (*n* = 1548)	*p*-Value
Age, years	73.83 ± 6.99	73.45 ± 7.34	0.002
Education, years	16.01 ± 2.85	15.84 ± 2.87	0.095
Sex, *n* (%)			0.43
Male	3277 (55.6)	832 (53.7)	
Female	2619 (44.4)	716 (46.3)	
*APOE* ε4 carrier, *n* (%)	2467 (41.8)	847 (54.7)	<0.001
ADAS-Cog13 cognitive score	17.40 ± 10.99	22.74 ± 11.53	<0.001
Aβ_42_/Aβ_40_ ratio	0.083 (0.077, 0.092)	0.080 (0.075, 0.086)	0.031

Continuous variables with normal distribution are presented as mean ± standard deviation, while non-normally distributed continuous variables are reported as median (interquartile range). Categorical variables are presented as *n* (%). Between-group comparisons were performed using the independent-samples t-test for normally distributed continuous variables, the Mann–Whitney U test for non-normally distributed continuous variables, and the χ^2^ test for categorical variables. Sample includes all participants with valid NPID sleep disturbance index data (*n* = 7444).

## Data Availability

The ADNI data used in this study are publicly available from the ADNI database (https://adni.loni.usc.edu). Access to ADNI data requires application and approval via the ADNI portal. No new raw data were generated in this study.

## Supplementary Materials

The following supporting information can be downloaded at: https://www.mdpi.com/article/10.3390/ijms27188271/s1.
