# Supplementary material for: Sleep Disturbance Correlates with Cognitive Impairment Partly via Plasma p-Tau217: ADNI Cross-Sectional Evidence and P301L Mouse Mechanistic Observations
Source: Int J Mol Sci. 2026 Sep 17;27(18):8271. doi: 10.3390/ijms27188271 (PMC13607299; doi:10.3390/ijms27188271)
Supplement: Supplementary file 1 [file ijms-27-08271-s001.zip › ijms-4499659-supplementary-Table S2.pdf]

Table S2. Variable Coding and Data Cleaning Definition

Data were derived from the Alzheimer's Disease Neuroimaging Initiative (ADNI) database. All data merging and preprocessing were performed following the standard procedures outlined below. We did not restrict analyses to baseline (VISCODE = bl) visits. Restricting exclusively to baseline visits would result in substantial sample loss due to sparse plasma p-Tau217 measurements at the baseline time-point. Instead, all valid visit-level observations with complete required variables were retained, where each record is uniquely identified by the combination of RID and VISCODE.

Variable Name	Source ADNI Table	Data Type	Coding & Value Definition	Data Cleaning & Exclusion Rules
RID	All merged tables	Character	Unique subject identifier, used as the exclusive matching key for merging all datasets	Retained in all steps; no missing RID allowed in the final merged dataset
NPID	NPI (Neuropsychiatric Inventory) table	Binary	Sleep disturbance (presence/absence)<br>0 = No sleep disturbance<br>1 = Presence of sleep disturbance	- Rows with NPID = -4 or NPIDSEV = -4 were deleted<br>- No screening by VISCODE (baseline only) was performed to avoid excessive sample size reduction
NPIDSEV	NPI (Neuropsychiatric Inventory) table	Ordinal	Sleep disturbance severity (for dose-effect analysis)<br>1 = Mild<br>2 = Moderate<br>3 = Severe	- Rows with NPIDSEV = -4 were deleted<br>- Retained only valid ordinal values
TOTAL13	ADAS-Cog table	Continuous	ADAS-Cog13 total score (cognitive function outcome)<br>Higher scores indicate more severe cognitive impairment	- Valid range: 0 ~ 85<br>- Values < 0 or > 85 were considered abnormal and excluded<br>- Rows with missing TOTAL13 were excluded
PTAU217	UPENN Plasma table	Continuous	Plasma p-Tau217 level	- Rows with missing PTAU217 were excluded<br>- Outliers outside the range of (mean ± 3SD) were excluded
Aβ42/40	UPENN Plasma table	Continuous	Plasma Aβ42/40 ratio	- Rows with missing Aβ42/40 were excluded
AGE	Demographics table	Continuous	Age at baseline visit<br>Calculated as: Visit Year - PTDOBYY	Derived from raw birth year (PTDOBYY) and visit year; no missing values allowed
PTGENDER	Demographics table	Binary	Raw gender code<br>1 = Male<br>2 = Female	Recoded to new variable GENDER for analysis

GENDER	Derived variable	Binary	Recoded gender for statistical analysis<br>1 = Male<br>0 = Female	No missing values allowed
PTEDUCAT	Demographics table	Continuous	Years of education (EDUC)	Used directly as a continuous variable; no missing values allowed
*APOE*4	*APOE* Genotype table	Binary	*APOE* ε4 carrier status<br>1 = ε4 carrier (genotypes: ε3/ε4, ε4/ε4)<br>0 = Non-carrier (genotypes: ε2/ε2, ε2/ε3, ε3/ε3)	- Genotypes other than the above were excluded<br>- Rows with missing/invalid genotype data were excluded
DIAGNOSIS	DXSUM_09Apr2026 diagnosis summary table	Categorical	ADNI standard diagnostic group<br>1 = CN (Cognitively Normal)<br>2 = MCI (Mild Cognitive Impairment)<br>3 = AD (Alzheimer's Disease)	- Subjects with diagnosis codes other than 1/2/3 were excluded<br>Used for stratified analysis across CN/MCI/AD groups
